# Supplementary material for: The Impact of Meal Dietary Inflammatory Index on Exercise-Induced Changes in Airway Inflammation in Adults with Asthma
Source: Nutrients. 2022 Oct 19;14(20):4392. doi: 10.3390/nu14204392 (PMC9610037; doi:10.3390/nu14204392)
Supplement: Supplementary file 1 [file nutrients-14-04392-s001.zip › nutrients-1952710-supplementary.pdf]

Table S1. Nutrients included in the calculation of E-DII.

Macronutrients		Vitamins		Minerals
Energy	Trans fat	Thiamin	Vitamin B12	Calcium
Fat total	Cholesterol	Riboflavin	Total folate	Iron
Saturated fat	Carbohydrate	Niacin	Folic acid	Magnesium
Monounsaturated fat	Sugar	Niacin equivalent	Vitamin A	Phosphorus
Polyunsaturated fat	Protein	Vitamin C	Retinol	Potassium
Omega 3	Fibre	Vitamin E	β-carotene	Selenium
Linoleic acid	Caffeine	α-tocopherol		Sodium
ALA	Alcohol	Vitamin B6		Zinc
				Iodine

ALA: alpha-linolenic acid.

Table S2. Correlation between the nutrients contained in the meal consumed two hours post-intervention with sputum eosinophil change at four hours.

Nutrient	Exercise		Control	
	Eosinophils (%)	Eosinophils (x10^4/mL)	Eosinophils (%)	Eosinophils (x10^4/mL)
Energy (kJ)	0.063	0.120	0.199	0.446
Fat (g)	0.010	0.107	-0.326	-0.096
Saturated fat (g)	-0.057	0.027	-0.268	-0.061
MUFA (g)	0.108	0.179	-0.245	-0.175
PUFA (g)	-0.054	-0.003	-0.187	-0.121
Long Chain Omega-3 (g)	-0.319	-0.294	-0.117	-0.350
ALA (g)	-0.021	0.066	-0.101	-0.046
Linoleic Acid (g)	-0.069	-0.019	-0.179	-0.193
Trans fat (g)	-0.020	0.050	-0.337	-0.082
Cholesterol (mg)	-0.252	-0.219	-0.129	-0.270
Carbohydrate (g)	0.037	0.053	**0.623***	0.482
Sugar (g)	0.172	0.232	0.375	**0.675***
Fibre (g)	0.181	0.196	0.251	0.361
Protein (g)	-0.074	0.015	0.038	0.325
Vitamins				
Vitamin A (µg)	-0.103	-0.035	-0.333	-0.274

Beta-carotene (µg)	0.055	0.133	-0.310	-0.271
Thiamin (mg)	0.040	-0.007	0.074	-0.339
Riboflavin (mg)	-0.194	-0.061	-0.225	-0.175
Niacin (mg)	0.053	0.043	0.021	0.371
Folic acid (µg)	0.351	0.303	0.097	-0.321
Vitamin B6 (mg)	-0.089	-0.033	0.251	**0.636***
Vitamin B12 (µg)	0.010	0.140	-0.415	-0.314
Vitamin C (mg)	0.045	0.018	-0.258	**-0.518***
Vitamin E (mg)	-0.217	-0.214	-0.177	-0.257
Minerals				
Calcium (mg)	-0.068	0.059	-0.052	0.057
Iron (mg)	-0.028	0.012	-0.173	-0.100
Magnesium (mg)	-0.000	0.138	0.090	0.393
Phosphorous (mg)	-0.013	0.029	0.136	0.371
Potassium (mg)	-0.009	0.033	0.162	0.157
Selenium (g)	-0.214	-0.179	0.054	-0.332
Sodium (mg)	0.290	0.314	0.376	0.243
Zinc (mg)	-0.095	-0.026	-0.221	-0.075

ALA: alpha-linolenic acid; MUFA: monounsaturated fatty acids; PUFA: polyunsaturated fatty acids. * $p<0.05$.

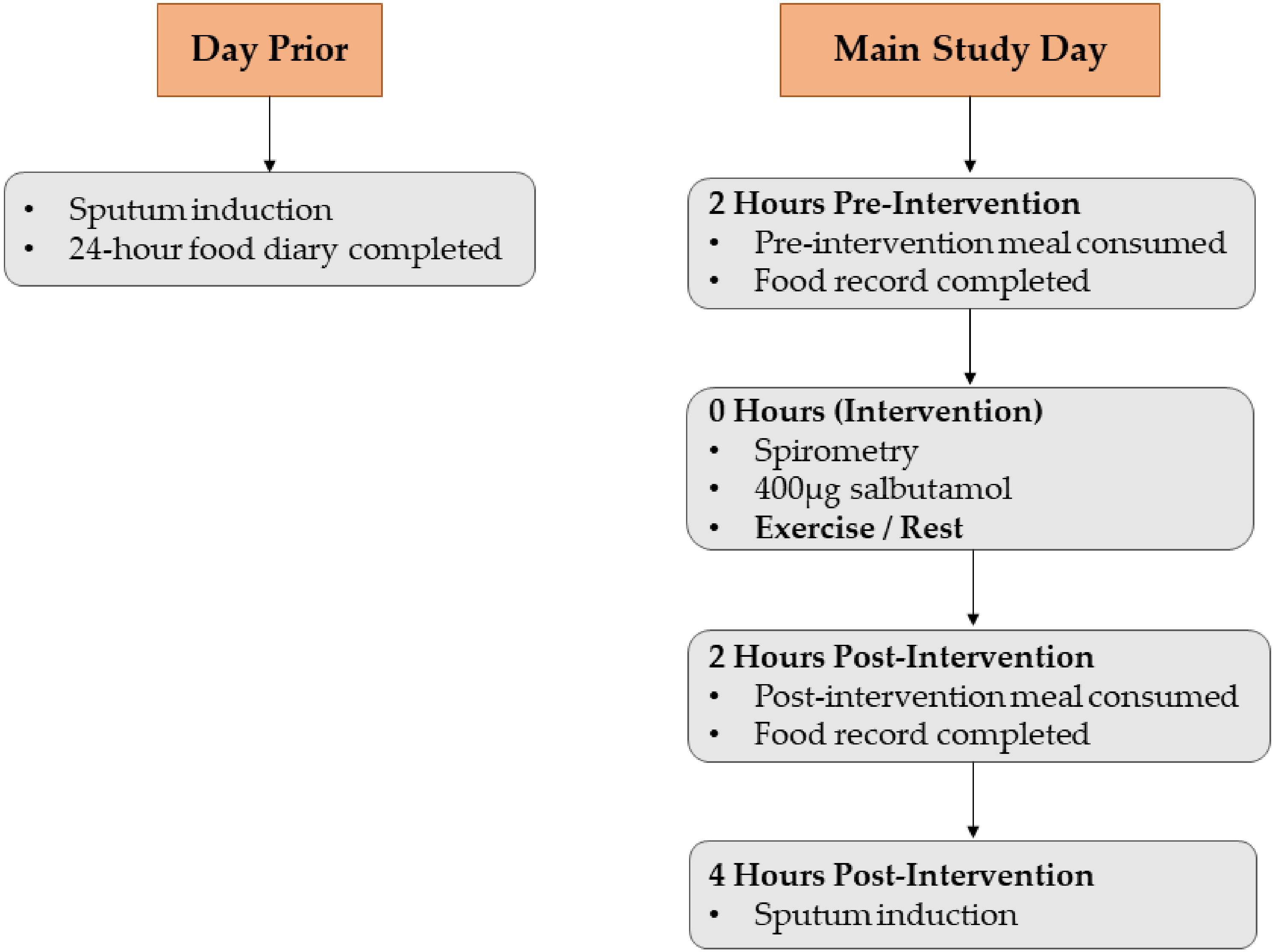

Figure S1: Flowchart outlining study procedures.
